# Liquid Crystal Modified Polylactic Acid Improves Cytocompatibility and M2 Polarization of Macrophages to Promote Osteogenesis

**DOI:** 10.3389/fbioe.2022.887970

**Published:** 2022-06-17

**Authors:** Zexiang Zheng, Renqin Wang, Jianjun Lin, Jinhuan Tian, Changren Zhou, Na Li, Lihua Li

**Affiliations:** ^1^ Department of Materials Science and Engineering, Engineering Research Center of Artificial Organs and Materials, Jinan University, Guangzhou, China; ^2^ Affiliated Stomatology Hospital of Guangzhou Medical University, Guangzhou Key Laboratory of Basic and Applied Research of Oral Regenerative Medicine, Guangzhou, China; ^3^ Foshan Stomatology Hospital, School of Medicine, Foshan University, Foshan, China

**Keywords:** liquid crystal, polylactic acid, cytocompatibility, macrophages, bone regeneration

## Abstract

Liquid crystalline phases (LC phases) are widely present in an organism. The well-aligned domain and liquidity of the LC phases are necessary for various biological functions. How to stabilize the floating LC phases and maintain their superior biology is still under study. In addition, it is unclear whether the exogenous LC state can regulate the immune process and improve osteogenesis. In this work, a series of composite films (PLLA/LC) were prepared using cholesteryl oleyl carbonate (COC), cholesteryl pelargonate (CP), and polylactic acid (PLLA) via a controlled facile one-pot approach. The results showed that the thermo-responsive PLLA/LC films exhibited stable LC phases at human body temperature and the cytocompatibility of the composites was improved significantly after modification by the LC. In addition, the M2 polarization of macrophages (RAW264.7) was enhanced in PLLA/LC films, and the osteogenic differentiation of bone marrow mesenchymal stem cells (BMSCs) was improved as co-cultured with macrophages. The *in vivo* bone regeneration of the materials was verified by calvarial repair*,* in which the amount of new bone in the PLLA-30% LC group was greater than that in the PLLA group. This work revealed that the liquid crystal-modified PLLA could promote osteogenesis through immunomodulation.

## 1 Introduction

Liquid crystal (LC) is a fluid with a micro-structure between an anisotropic crystal and an isotropic liquid, also known as a mesogenic or LC phase which has remarkable viscoelastic characteristics (Lowe and Abbott, 2012; Soon et al., 2013; Du et al., 2017; Chaudhuri et al., 2020). The LC phase exists in different organisms, such as in their bone (Giraud-Guille et al., 2000; Hulmes, 2002; Ghazanfari et al., 2016), chitin (Li et al., 2019; Liu et al., 2020), nucleic acids (Saurabh et al., 2017), lipids (Róg et al., 2009), and so on (Stewart, 2004; Rey, 2010; Hirst and Charras, 2017). Physiological activities such as mass transport, information transmission, cell recognition, and cell fusion and division cannot proceed smoothly without the LC phase of the cell films (Jewell, 2011; Gupta et al., 2015; Wu et al., 2017; Saw et al., 2018; Chen et al., 2020). Different LC elastomers have been studied and showed unique longitudinal and multi-responses for cell attachment, proliferation, and alignment (Prévôt and Hegmann, 2017). For example, LC collagen made by concentrated solution and aligned shearing was proved to possess good guidance for cell attachment and proliferation (Price et al., 2016; Tang et al., 2016). Cholesterol is an important component of cell films and has a large affinity for cells, so it has been widely studied and used in the biomimetic design of biomaterials (Li et al., 2001; Hwang et al., 2002; Chen et al., 2020). However, the mixing of cholesterol with other materials always causes spontaneous phase separation and leads to the rapid aggregation of cholesterol. This will result in high cholesterol concentration and cytotoxicity. Previous studies showed that cholesteryl ester ramification was a new class of bio-related materials with good thermal stability and low toxicity (Soon et al., 2009; Soon et al., 2014). In the literatures, cholesteryl ester ramification is usually applied solely to modify polymeric biomaterials to get higher cellular affinity (Li et al., 2001; Du et al., 2017). However, most biocompatible cholesteryl substances such as COC and CP with good blood compatibility are not in the LC state at the range of human physiological temperatures, which block the biological function of the LC phase in the human body (Li et al., 2001; Xie et al., 2020). According to the well-known van’t Hoff equation, the transition temperature (clean point) of the composite liquid crystals is lower than that of each component. But in most of the cases, the measured clean point sits between that of each component (Tai and Lee, 1990). Hence, the clean point of the CP/COC mixture could be near 37°C by controlling their concentrations, because the clean point for CP and COC is 90 and 35°C, respectively.

PLLA has multiple distinguished features, including easy processing, good thermal stability, excellent mechanical and physical properties, and biodegradability in the human body of which degradation products have no side effects (Agrawal and Ray, 2001; da Silva et al., 2018; Dhandapani et al., 2020). Therefore, PLLA is approved by the FDA as the type III medical material and has been widely used in the field of medicine (Madhavan Nampoothiri et al., 2010; Yao et al., 2017; Wei and Dai, 2021). However, PLLA lacks active functional groups for cell adhesion, spreading, and proliferation, which leads to unsatisfactory cytocompatibility. In addition, the acidic degradation products will cause inflammation and then hinder the regeneration of tissues when the PLLA is used as bone repair material (Bergsma et al., 1993; Critchley et al., 2020).

It is well known that bone repair is, in fact, involved in the process of immune regulation (Schlundt et al., 2015; Chen et al., 2016; Lee et al., 2019). Macrophages are one of the cells that play important roles in regulating the inflammatory response (Browne and Pandit, 2015), which can be polarized into pro-inflammatory M1 macrophages or anti-inflammatory M2 macrophages under the stimulation of the inflammatory environment (Freytes et al., 2013; Spiller et al., 2015; Zhang et al., 2017; Huang et al., 2020; Mahon et al., 2020; Qiu et al., 2020). Once the biomaterials are implanted into the body, a series of inflammatory responses will be activated. To date, researchers have been trying to improve cell compatibility and immunogenicity of PLLA through bulk or surface modification, to introduce specific biological functional ligands (Lutolf and Hubbell, 2005; Liu et al., 2017; Pellegrino et al., 2017; Liu et al., 2019) or biologically active molecules (Ding et al., 2016; Li X et al., 2021).

In view of the good biocompatibility and suitable LC state temperature of CP and COC, PLLA was modified by blending the LC state to enhance its functionality, and to study the immunoregularity of the composite materials for bone regeneration. Since CP, COC, and PLLA are easily dissolved in organic reagents and form uniform composite by solution casting, a series of functional PLLA/LC films were obtained by a one-pot but controlled blending approach. We expected that the bionic liquid crystal composite materials would better simulate the microenvironment of tissue culture *in vivo*, including the compatibility of PLLA materials and regulation of inflammatory response for tissue regeneration. Many exciting anti-inflammatory results of the liquid crystal materials were observed in our preliminary experiments.

## 2 Materials and Methods

### 2.1 Materials

Polylactic acid (PLLA, Mw = 200000, PDI = 1.5, CAS: 33135-50-1) was provided by Jinan Daigang Biomaterials Co., Ltd. Cholesteryl oleyl carbonate (COC, CAS: 17110-51-9) was purchased from Tokyo Chemical Industry. Cholesteryl pelargonat (CP, CAS: 1182-66-7) was purchased from Sigma-Aldrich. Dulbecco’s Modified Eagle Medium (DMEM), fetal bovine serum (FBS), and penicillin–streptomycin solution were purchased from Gibco BRL. The Cell Counting Kit-8 (CCK-8) was purchased from Dojindo Molecular Technologies Inc. The AO-EB Double Staining Kit was purchased from Beijing Solarbio Science and Technology Co., Ltd. Rhodamine phalloidin and 4′, 6-dimidyl-2-phenylindole (DAP, CAS:28718-90-3) were purchased from Molecular Probes, United States. Osteoblastic induction medium and Alizarin Red solution were purchased from Cyagen Biosciences Inc. Fluorescein isothiocyanate conjugated anti-mouse CD206 (FITC-CD206) and Allophycocyanin conjugated anti-mouse CD80 (APC-CD80) were purchased from Dakewe Biotech Co., Ltd. The BCIP/NBT alkaline phosphatase color reagent kit was purchased from Beyotime Biotechnology Co., Ltd. ALP and the BCA protein assay kit was purchased from Nanjing Jiancheng Bioengineering Institute. All cells were obtained from the American Type Culture Collection (ATCC). All other reagents were purchased from Guangzhou Reagent Co., Ltd., analytical grade.

### 2.2 Preparation of COC/CP Composite LC

A series of COC/CP composite liquid-crystal were prepared by the melting method. In brief, a certain amount of COC and CP was added into a 5 ml centrifuge tube at room temperature. The tube was transferred to an oven at 90 °C. The liquid COC and CP were then mixed by stirring. The COC weight fraction in the composite LC was 0–100% with a gradient of 10%.

### 2.3 Preparation of PLLA/LC Composite Film

Three different PLLA/LC composite films were prepared by the solution casting method. According to our previous work (Li N et al., 2021), PLLA and LC (70%COC-30%CP) were stirred in dichloromethane at room temperature for 6 h to produce a clear and uniform PLLA/LC solution. The solution was then cast on a clean Teflon plate and the solvent was evaporated in a clean environment for 48 h. Then the film was placed in a vacuum oven for 12 h to remove the residual solvent. The weight fractions of the LC in the composite films were 10%, 30%, and 50%, which were labeled as PLLA-10% LC, PLLA-30% LC, and PLLA-50% LC, respectively. The thickness of the film and the aggregation state of the LC in the composite film could be controlled by the properties of the solvent, the concentration of the solution, and the temperature.

### 2.4 Thermal Analysis

A differential scanning calorimeter (DSC) (TA, Netzsch, Germany) was used to measure the clean point and the liquid-crystal phase transition temperature of COC/CP LC and PLLA/LC composite films. The heating and cooling rate was 10°C min^−1^ while the scanning ranges were −20–100°C and 0–200°C for COC/CP LC and PLLA/LC, respectively. The secondary temperature rise curve was taken for analysis.

The LC phase texture of the COC/CP LC and PLLA/LC composite film was observed by using polar optical microscope (POM, OLYMPUS, Japan). The slide was placed on the hot platform, a small amount of sample was placed on the slide, and the cover glass was pressed. The heating and cooling rates were set as 10°C·min^−1^ by a heating and cooling program.

### 2.5 Cytocompatibility Analysis

#### 2.5.1 Cell Culture

In this experiment, mouse pre-osteoblasts (MC3T3-E1) were selected as seeded cells to evaluate the cytocompatibility of the materials *in vitro*. The cell culture medium was a complete medium containing 10% fetal bovine serum, 1% antibiotics, and 89% DMEM. The cells were cultured in a T25 culture flask in a CO_2_ incubator at 37°C, 5% CO_2_ concentration, and 95% relative humidity. The cell culture medium was replaced every other day.

The LC-10% CP, LC-30% CP, LC-50% CP, PLLA, PLLA-10% LC, PLLA-30% LC, and PLLA-50% LC composite films were immersed in 75% ethanol for 1 h and sterilized under UV for 2 h. The cells were seeded on different films at a density of 1×10^4^ cells·well^−1^ in three parallel samples per group after sterilizing.

#### 2.5.2 Cell Activity and Toxicity Analysis

A CCK-8 kit was used to evaluate MC3T3-E1 cell activity at 1, 3, 5, and 7 days, the medium in the wells was aspirated and the cells were washed once with PBS. A CCK-8 reaction solution was used right after it was prepared from the CCK-8 solution and fresh complete medium at a volume ratio of 1:10. A total of 500 μL reaction liquid was added to the 24-well plate and placed in a CO2 incubator and incubated in the dark for 2 h. From each sample well, 100 μL of the reaction solution was aspirated into a 96-well plate. The OD value of each well was measured by enzyme-linked immunoassay at λ_max_ = 450 nm.

The cells were stained with AO-EB solution to evaluate the toxicity of the material. At 1 and 3 days, the cells were washed twice with PBS, followed by AO-EB staining (AO: EB: PBS = 1: 1: 100) for 5 min at 37°C. After staining, the cells were washed again with PBS and observed under a fluorescent microscope. Living cells were stained green by acridine orange (AO) and dead cells were stained red by ethidium bromide (EB).

#### 2.5.3 Cell Morphology and Microstructure

When the cells were cultured on the material for 3 days, the medium in the well was aspirated, the cells were washed twice with PBS, and a 4% paraformaldehyde solution was added to fix the cells for 30 min. Then, the materials were soaked in a series of gradient concentration ethanol solutions (volume ratio: 50%, 60%, 70%, 80%, 90%, 95%, 100%) for 15 min. The samples were air-dried at room temperature and sprayed with gold, and then were observed and photographed under SEM (ULTRA55, Carl Zeiss Jena Ltd, German) with 5 kV acceleration voltage under a scanning electron microscope.

The samples were washed with PBS and fixed with 4% paraformaldehyde for 20 min. Then they were washed with PBS three times and permeabilized with 0.1% Triton X-100 for 5 min. The cells were blocked by adding 3% BCA solution for 30 min, washed three times with PBS, and incubated with rhodamine phalloidin for 1 h. They were washed twice with PBS after incubating with DAPI for 15 min at room temperature. Cytoskeleton analysis was then performed following the steps described previously.

#### 2.5.4 Cell Migration Experiment

LC-30% CP was coated on 50% of the 14-mm diameter cell slide. The samples were then placed in a confocal Petri dish and sterilized by UV for 3 h. The cells were seeded on the materials at a density of 5×10^3^ cells·well^−1^ in three parallel samples per group on 24-well culture plates.

The films were transferred into 24-well plates with a 600 μL culture medium. With transwell chambers (pore size = 8 μm), 100 μL of cell suspension was added to each sample with 5×10^3^ cells. The cells were stained with crystal violet after culturing for 24 and 72 h. Then the chambers were observed under a microscope and pictures were taken, and randomly selected three visual fields under the appropriate magnification microscope to observe the cell number and count. The stained cells on the chambers after 72 h were decolorized with 33% acetic acid. The decolorization solution was transferred into a 96-well plate with 100 μL per well. The OD values were measured at 570 nm.

### 2.6 Osteogenic Immunomodulation

#### 2.6.1 Polarization of Macrophages

A mouse macrophage cell, RAW264.7, was selected in this experiment. The cells were seeded into 24-well plates with different prepared films at a density of 1×10^4^ cells·well^−1^. The cells were collected after culturing for 24 and 48 h. After adding FITC-CD206 and APC-CD80 (FITC-CD206:APC-CD80: PBS = 1:1:100), the cells were incubated for 30 min at 4°C in the dark. The polarization of RAW264.7 was analyzed by using an analytical flow cytometer (BD FACSCanto, America).

#### 2.6.2 Alkaline Phosphatase (ALP) Staining and Quantitative Analysis

RAW264.7 and bone marrow mesenchymal stem cells (BMSCs) were co-cultured with an osteoblastic induction medium in this experiment. A total of 600 μL of BMSCs was seeded into 24-well plates with different prepared films at a density of 1×10^4^ cells·well^−1^. The transwell chambers (pore size = 0.4 μm) were put into plates. A total of 100 μL of RAW264.7 suspension was added to each chamber at a density of 1×10^4^ cells·well^−1^. The plates were put into the incubator and culture for 2 days, and then the chambers were taken out. After 7 days of culture, the BMSCs were stained with a BCIP/NBT alkaline phosphatase color reagent kit. After 7 and 14 days of culture, an alkaline phosphatase kit and BCA protein kit were used to detect the alkaline phosphatase activity of the BMSCs.

#### 2.6.3 Alizarin Red Staining and Quantitative Analysis

RAW264.7 and bone marrow mesenchymal stem cells (BMSCs) were co-cultured with an osteoblastic induction medium following the procedure 2.6.2. After 21 days of incubation, the samples were washed with PBS and fixed with 4% paraformaldehyde for 20 min. After washing with PBS three times, the samples were stained with Alizarin Red solution and observed under an optical microscope. After imaging, 500 μL of 10% hexadecylpyridinium chloride monohydrate was added to each well. After 30 min, 100 μL of the reaction solution from each well was aspirated into a 96-well plate. The OD value of each well was measured by an enzyme-linked immunoassay at λmax = 540 nm. The control group in Figures 5, 6 means that the cells were growing on the 24-well plates.

#### 2.6.4 Polymerase Chain Reaction CR Analysis

RAW264.7 and bone marrow mesenchymal stem cells (BMSCs) were co-cultured with an osteoblastic induction medium using the procedures presented in 2.6.2. After 7 and 14 days of culture, the cells were collected. The gene expression of the osteogenic related genes OCN, COL-1, OPN, and Runx-2 of the BMSCs cultured on different films were detected by RT-PCR technology, using β-actin as an internal control. The PCR primer series is shown in Table 1.

**TABLE 1:** Osteogenic primer sequences for RT-PCR.

Gene	Forward sequence (5′-3′)	Reverse sequence (3′-5′)
GAPDH	GGC​CTC​CAA​GGA​GTA​AGA​AA	GCC​CCT​CCT​GTT​ATT​ATG​G
OCN	AGG​GCA​ATA​AGG​TAG​TGA​A	CGT​AGA​TGC​GTC​TGT​AGG​C
COL-Ι	CTT​CAC​CTA​CAG​CAC​CCT​TGT	AAG​GGA​GCC​ACA​TCG​ATG​AT
OPN	ACC​ATT​CGG​ATG​AGT​CTG​AT	TCA​GTC​CAT​AAG​CCA​AGC​TA
Runx-2	TGC​CCA​GTG​AGT​AAC​AGA​AAG​AC	CTC​CTC​CCT​TCT​CAA​CCT​CTA​A

### 2.7 Rat Skull Defect Repair Experiment

Female Sprague–Dawley (SD) rats were selected to construct a skull defect model as control, PLLA, and PLLA-30% LC groups. Each group had three rats with a weight of 220–280 g. All the rats were purchased from the Experimental Animal Center of Southern Medical University. The rats were anesthetized with 1% pentobarbital sodium (30 mg/kg). After anesthetizing, the heads of the rats were disinfected with 75% alcohol and iodophor. A 2.5-cm incision was made vertically in the center of the rat’s head. A 5-mm diameter ring drill was used to make a 5-mm hole on both sides of the center of the parietal bone with full thickness. PLLA-30% LC films were filled into the bone defect. The periosteum, subcutaneous tissue, and skin were then sutured in layers. After 4 weeks, the rats were sacrificed by cervical dislocation. The collected skulls were fixed with 4% paraformaldehyde for subsequent micro-CT detection and H&E staining.

### 2.8 Statistic Analysis

All results are reported as mean ± standard deviation and statistically analyzed using Graphpad Prism 7.0 software. At least three parallel samples (n = 3) of each group were used for all experiments. This value is considered statistically significant only when *p* < 0.05.

## 3 Result and Discussion

### 3.1 Thermal Properties of COC/CP Liquid-Crystal and PLLA/LC Composite Films

**FIGURE 1:**
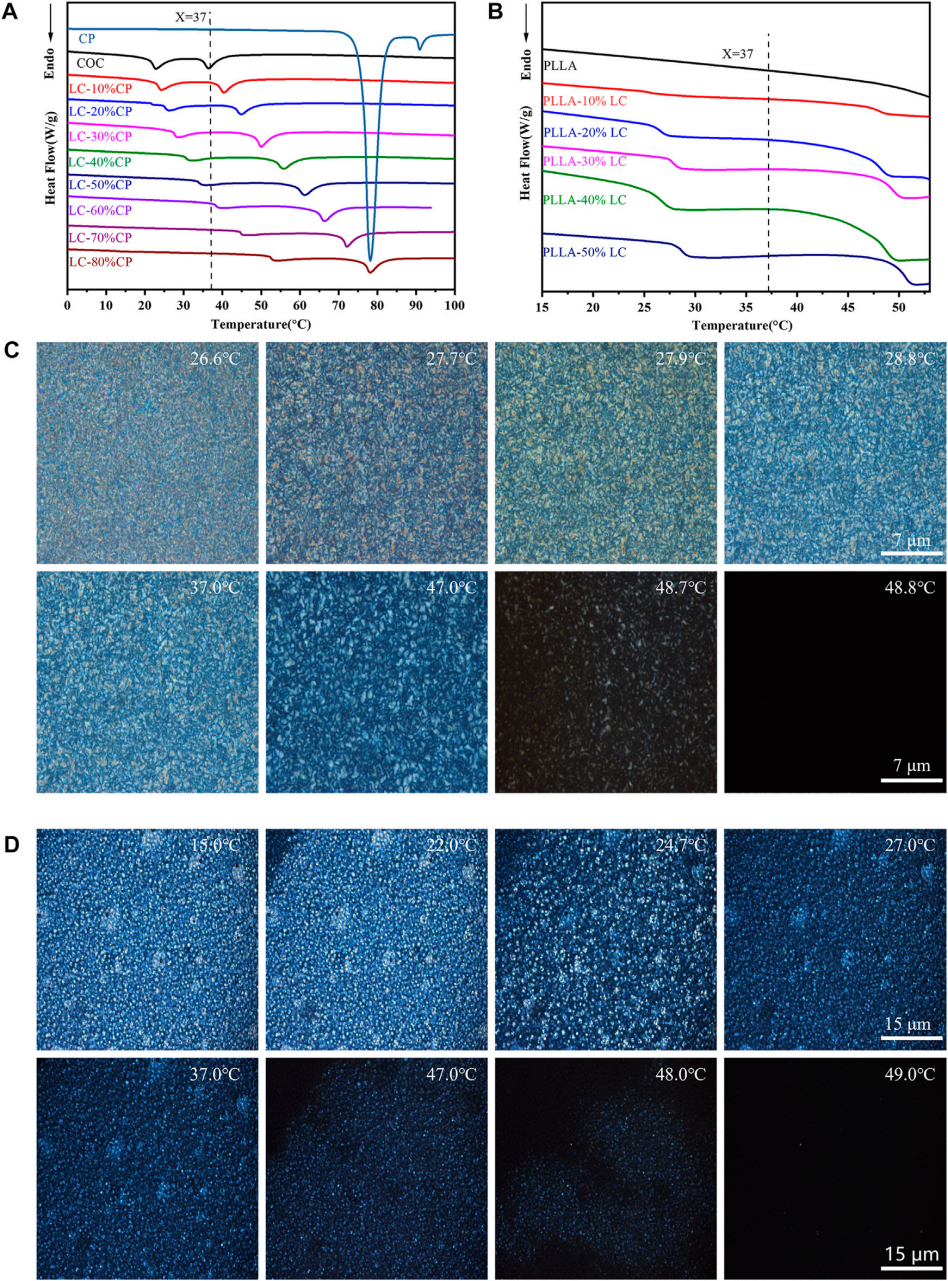
Thermal properties of the composite LC. **(A)** DSC curve of COC/CP LC; **(B)** DSC curve of PLLA/LC composite films; **(C)** blended LC LC-30% CP LC texture observed under POM; and **(D)** LC texture of the PLLA-30% LC composite films observed under POM.

According to DSC thermal analysis, no LC phase was detected in pure COC or CP (Figure 1A) at 37°C. However, by blending COC and CP, the cholesteric ester LC phase was obtained at 37°C (Figure 1A). It was worth mentioning that the mixture (LC-30% CP) indicated good stability and 37°C was the middle point of the phase transition temperature range. The liquid-crystal phase transition temperature of the COC/CP liquid-crystal measured by DSC matched the measurement in POM. At 37°C, an obvious LC phase flow could be seen (Figure 1C). With the increase in the CP concentration, the liquid-crystal phase transition temperature of COC/CP also increased. The LC phase transition temperature became higher than 37°C when the CP concentration exceeded 50%. The increase in the LC phase transition temperature of the blend is because the increase in CP (decrease of molecular dimensions) caused the increase in the phase-transition temperature of the COC/CP binary system (Li N et al., 2021). The clearing point of the mixed LC followed the empirical formula (Tai and Lee, 1990):

$$\frac{1}{T_{i}}=\frac{W_{cp}}{T_{cp}}+\frac{W_{coc}}{T_{coc}},$$ (1)

*T*_*i*_ is the clear point of the mixture, *W*_*cp*_ is the weight fraction of CP in the mixture, *T*_*cp*_ is the clearness of CP, *W*_*coc*_ is the weight fraction of COC in the mixture, and *T*_*coc*_ is the clearness of COC in the formula.

By adding COC/CP LC, an LC phase could be formed within PLLA. The LC phase was uniformly distributed in the composite film at 37°C (Figure 1B, D). This liquid crystal phase domain could promote the transport of materials and the transmission of signals. It also enabled the response to external temperature stimuli which successfully simulated the flow mosaic model of biofilms (Wu et al., 2017; Chen et al., 2020).

### 3.2 Cell Viability

**FIGURE 2:**
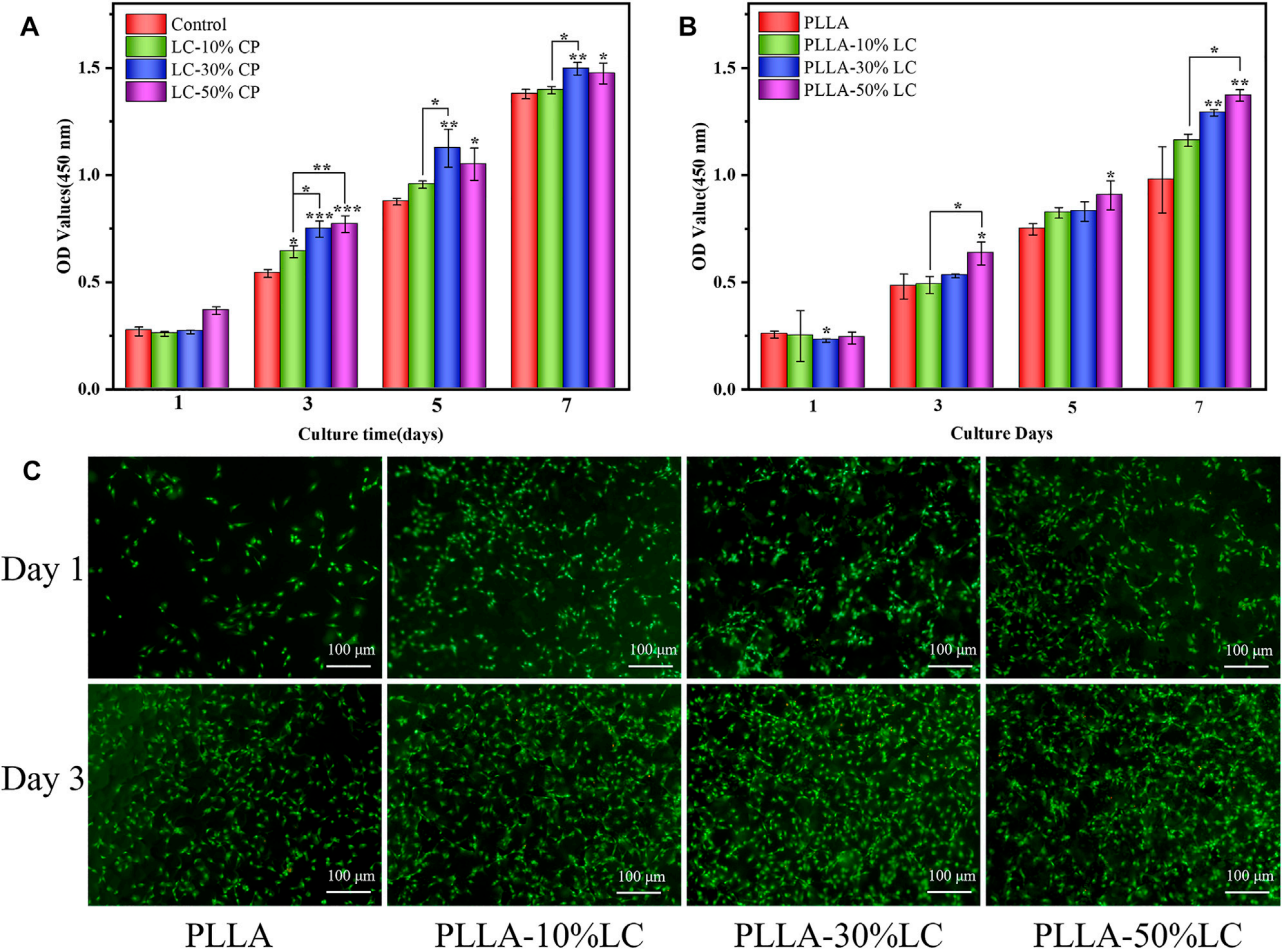
**(A)**Cell proliferation in COC/CP LC and **(B)** PLLA-LC composites for 7 days * refer to statistically significant proliferation *p* < 0.05. **(C)** AO-EB staining of MC3T3-E1 cells seeded on pure PLLA, PLLA-10% LC, PLLA-30% LC, and PLLA-50% LC films.

CCK-8 and live/dead assays were carried out to evaluate the cytocompatibility of the LC materials using MC3T3-E1. Compared with the control group, COC/CP LC had better cytocompatibility while LC-30% CP showed the best compatibility (Figure 2A). This was also why LC-30% CP was chosen to modify PLLA in subsequent studies. The MC3T3-E1 cell proliferation rate was significantly higher than the controlled group when the concentration of LC in the PLLA/LC composite film was 30% (wt) and 50% (wt) (Figure 2B). The cell density on the PLLA/LC composite films was greater than that on the PLLA film (Figure 2C) indicating that the LC materials were non-cytotoxic, and the addition of the LC facilitated cell proliferation. This was because many life processes associated with cell proliferation such as mass transport, information transmission, cell recognition, and cell fusion and division can proceed smoothly because of the flowing LC state (Wu et al., 2017; Saw et al., 2018; Chen et al., 2020).

### 3.3 Cell Morphology

**FIGURE 3:**
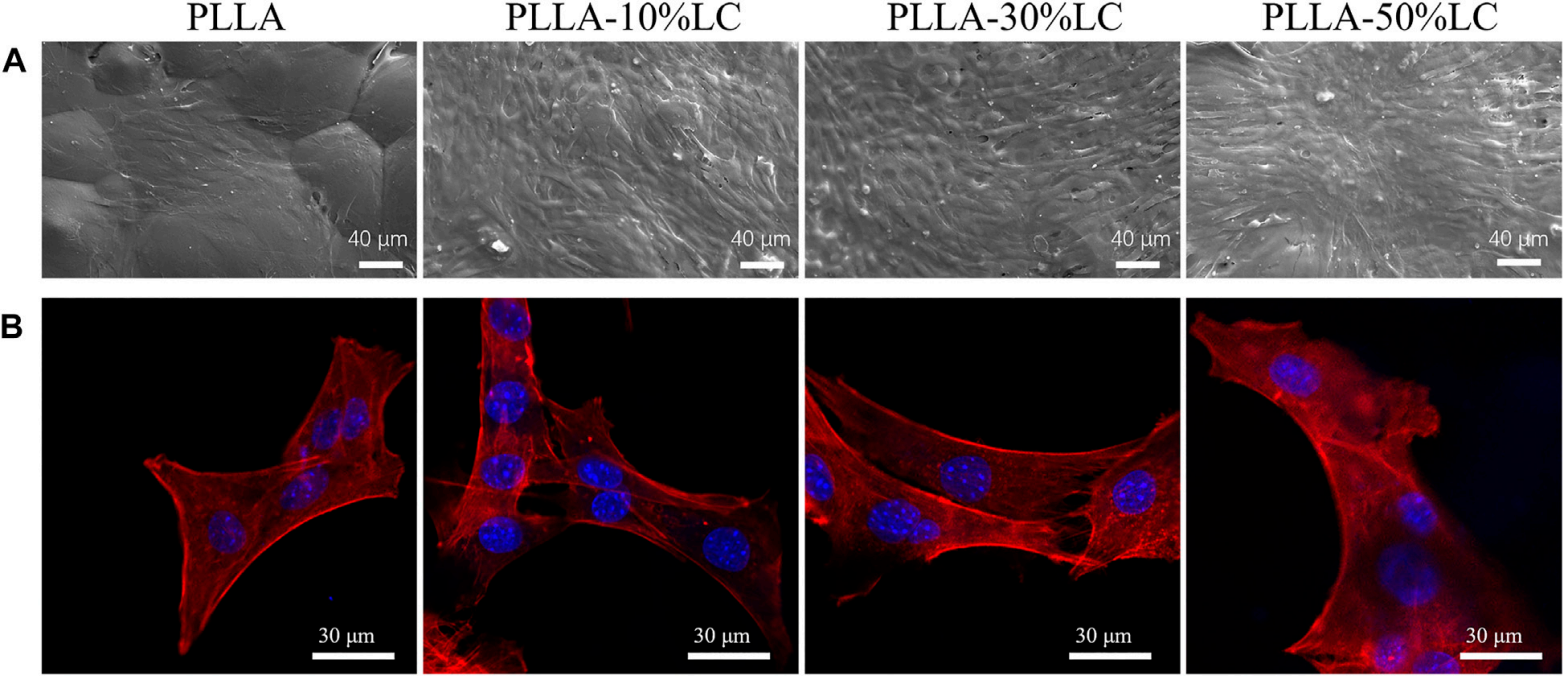
**(A)**SEM images of morphologies of MC3T3-E1 cells culturing on pure PLLA, PLLA-10% LC, PLLA-30% LC, and PLLA-50% LC films for 3 days; **(B)** CLSM images of morphologies of MC3T3-E1 cell culturing on pure PLLA, PLLA-10% LC, PLLA-30% LC, and PLLA-50% LC films for 1 day.

Compared with pure PLLA, MC3T3-E1 cells could adhere tighter to the PLLA/LC composite films and yield more ECM which was confirmed by SEM (Figure 3A). The cells connected to each other through ECM suggesting that cells showed better viability on the surface of PLLA/LC composite films. From the laser confocal microscope (CLSM) photo (Figure 3B), compared with pure PLLA, MC3T3-E1 cells fully covered the surface of PLLA/LC composite films with well-extended actin fiber bundles and filamentous pseudopods. Moreover, the nucleus structure was complete, and the cytoplasm was stained uniformly. Some cells were in contact with each other, reflecting an irregular or polygonal shape. We speculated that it was the LC environment that helped the cell to guide and guided the flow and order of the LC to guide the extension of the cell’s pseudopods. According to SEM and CLSM, MC3T3-E1 cells exhibited better early adhesion and growth on the surface of PLLA/LC than pure PLLA. This observation implied that the cholesteric LC in the PLLA matrix can still exert its bioactive effect. It was further confirmed that LC concentrations of 30% (wt) and 50% (wt) improved cell adhesion of PLLA.

### 3.4 Cell Migration

**FIGURE 4:**
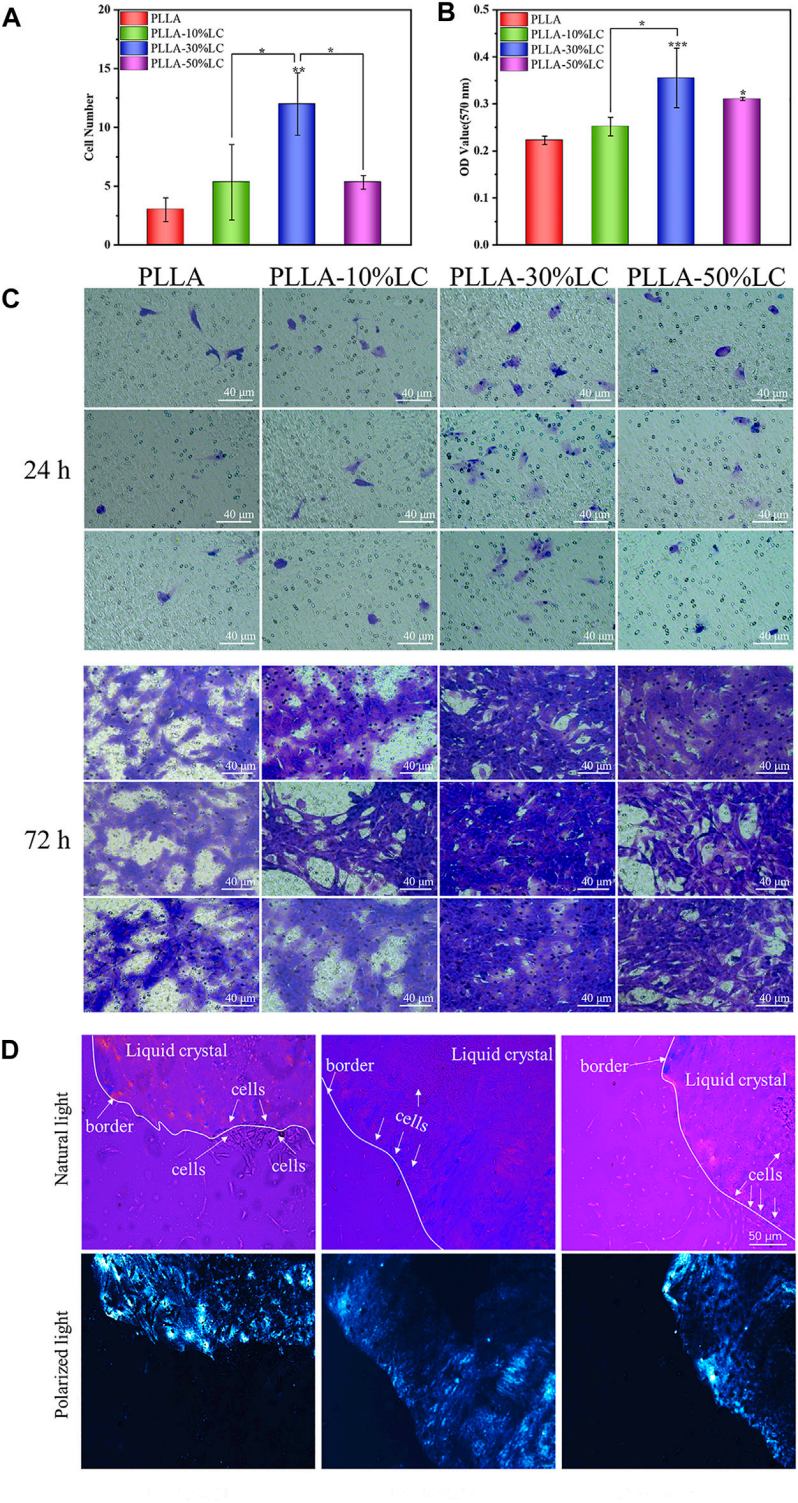
**(A)** Cell count of osteoblasts migrating in the transwell chamber for 24 h; **(B)** quantitative analysis of osteoblasts migrating in the transwell chamber for 72 h; **(C)** crystal violet staining of osteoblasts migrating in the transwell chamber for 24 and 72 h; and **(D)** distribution of cells on LC-30% CP.

The cell migration experiment (Figure 4A–C) showed that MC3T3-E1 cells had a higher migration rate in PLLA/LC than in the cell slide and pure PLLA. Especially in the PLLA/LC (30% wt) composite film, the numbers and spreading effect of MC3T3-E1 cells were much higher than pure PLLA at 24 and 72 h. This confirmed that COC/CP cholesterol esters could significantly increase the cell affinity of PLLA.

From the POM images, the boundary between the liquid crystal and the non-liquid crystal area (the black part in the picture) was obvious. Figure 4D indicates that the cells were likely to spread out within the liquid crystal domains rather than the glass matrix. The cell density of different areas was different, and there were obvious boundaries (Figure 4D). This boundary just coincided with the distribution boundary of the cells, which may be due to the LC phase domains of the LC mimicking the movable morphology of the natural biomembrane surface which probably provided active sites for cell attachment to the substrate and then promote cell adhesion.

### 3.5 Osteogenic Immunomodulation

**FIGURE 5:**
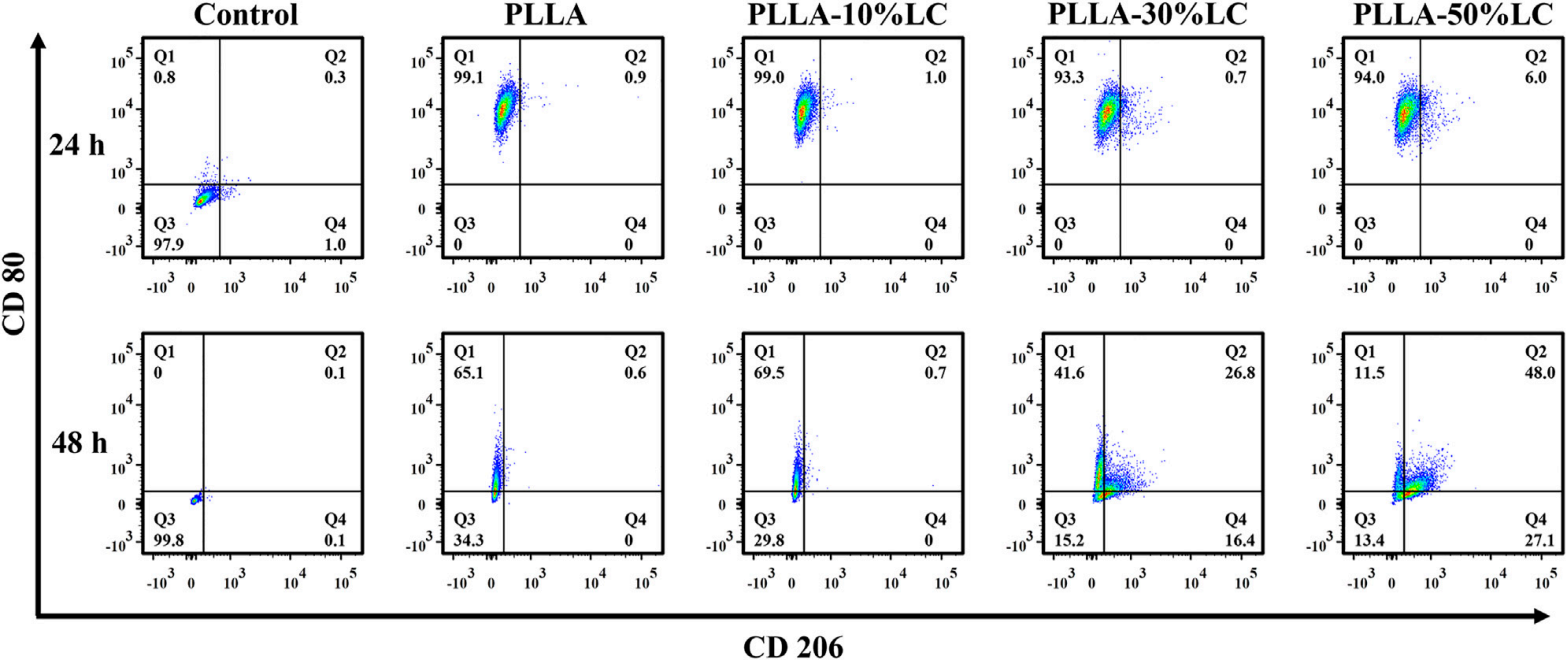
Polarization of macrophages on the material.

The results of the macrophage polarization experiment (Figure 5) indicated that the macrophages highly expressed CD80 which is mainly pro-inflammatory M1 macrophages after being cultured for 24 h on both PLLA and PLLA/LC films. However, the expression of CD206 of macrophages was increased with the increase of LC-30% CP content after being cultured 48 h with PLLA-30% LC and PLLA-50% LC films. This indicated that the PLLA/LC films can significantly promote M2 polarization of RAW264.7 while the liquid crystal content reaches a certain height. It is worth mentioning that the biological effect caused by LC domains could reduce the inflammatory response of the material implanted in the organism during bone tissue repair, which was beneficial to the reparation of bone tissue (Schlundt et al., 2015; Chen et al., 2016; Lee et al., 2019).

**FIGURE 6:**
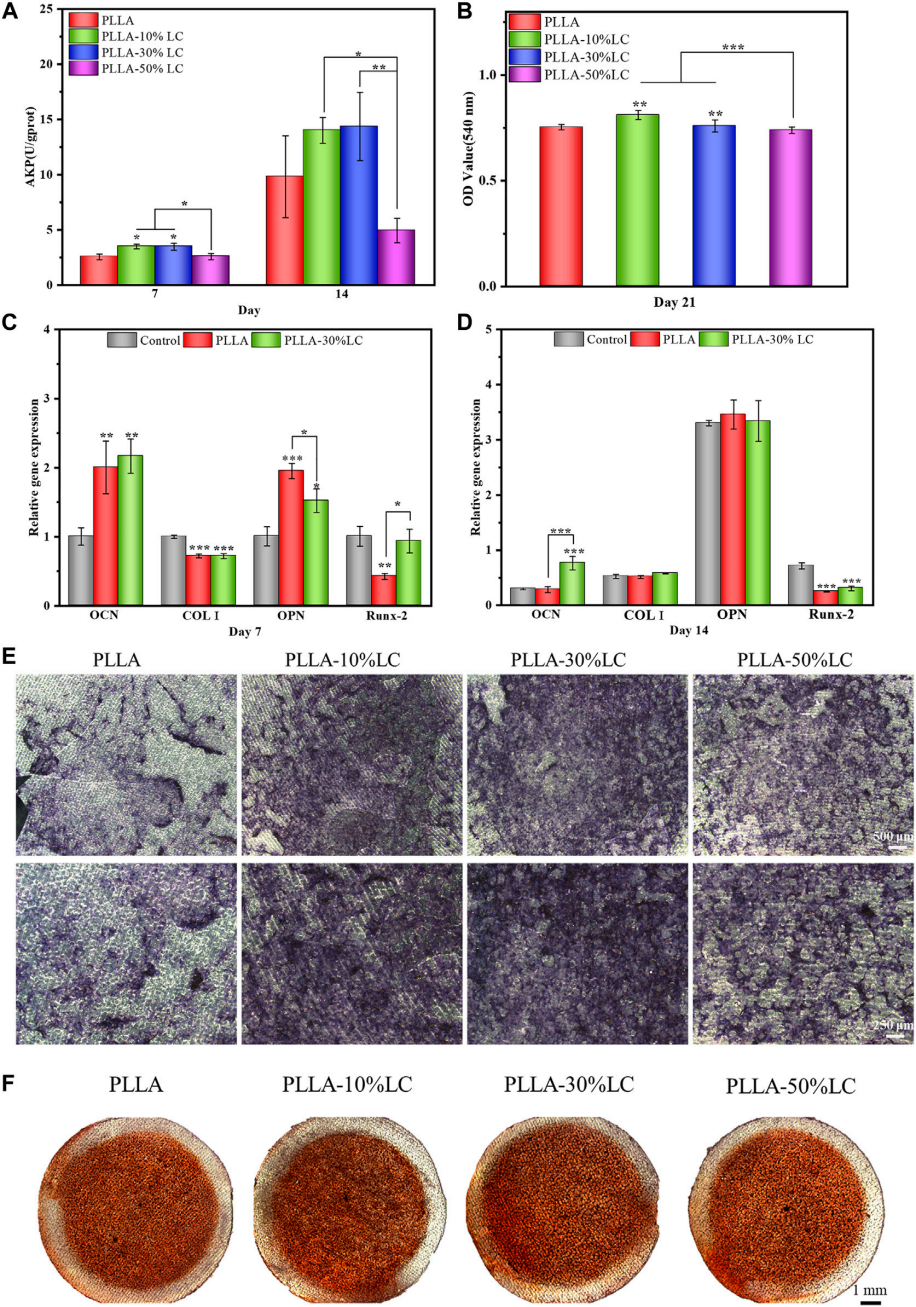
**(A)** ALP quantitative analysis; **(B)** Alizarin Red staining quantitative analysis; **(C)** and **(D)** BMSC-related osteogenic gene expression on different materials; **(E)** ALP staining; and **(F)** Alizarin Red dyeing.

Bone tissue repair involved a series of complicated dynamic immune regulatory processes. To simulate the microenvironment of bone tissue repair in the living body more realistically, the macrophages were co-cultured with BMSCs to observe the osteogenic differentiation of BMSCs under a corresponding immune condition environment. ALP is a specific protein secreted in the early stage of osteogenic differentiation. The ALP staining results (Figure 6E) showed that PLLA-10% LC and PLLA-30% LC composite films could significantly promote the ALP expression of BMSCs compared with PLLA on the 7th day, which was consistent with the result of the ALP quantitative analysis (Figure 6A). However, the ALP expression of BMSCs cultured in PLLA-50% LC composite films was lower than that of PLLA whether it was on the 7th or 14th day, which also indicated that the liquid crystal proportion would not linearly generate PLLA/LC composite films to promote the osteogenic differentiation of BMSCs.

In the late stage of osteogenic differentiation, BMSCs deposit calcium and phosphate salts forming calcium nodules. Calcium nodules could be used to evaluate the degree of osteogenic differentiation of BMSCs, for they can chelate with Alizarin Red and form red insoluble. Quantitative analysis of calcium nodules was usually used to evaluate the late stage of osteogenic differentiation of BMSCs, since it was considered a benchmark to evaluate the bone induction ability of materials. It could be seen from Figure 6F that the calcium nodules on the PLLA-50% LC group were similar to the PLLA group, and the calcium nodules were lighter in red which indicated that the number of calcium nodules on the surface was smaller. The calcium nodules in PLLA-10% LC and PLLA-30% LC groups were significantly darker than those in the PLLA group, which indicated that the number of calcium nodules was significantly higher in PLLA-10% LC and PLLA-30% LC groups. Similarly, the quantitative analysis of calcium nodules (Figure 6B) showed that the OD values of PLLA-10% LC and PLLA-30% LC groups were higher than those in the PLLA and PLLA-50% LC groups. The OD value of the PLLA-10% LC group was basically the same as that of the PLLA-30% LC group. The result of the calcium nodule quantitative analysis further proved that the bone formation performance of PLLA-10% LC and PLLA-30% LC groups was significantly better than in the other groups.

The PCR results (Figure 6C, D) showed that on the 7th day, the expression of Runx-2 of BMSCs cultured in the PLLA-30% LC film was significantly higher than that of PLLA. On the 14th day, the expression of OCN of BMSCs in PLLA-30% LC films was significantly higher than in PLLA. Runx-2 is a sign of the beginning of osteogenic differentiation of cells, and OCN is a sign of osteogenic differentiation and maturation of cells (Schroeder et al., 2005; Chen et al., 2016). These results further showed that PLLA-30% LC films could better enhance the osteogenic differentiation of cells than PLLA.

### 3.6 Calvarial Repair *In Vivo*

**FIGURE 7:**
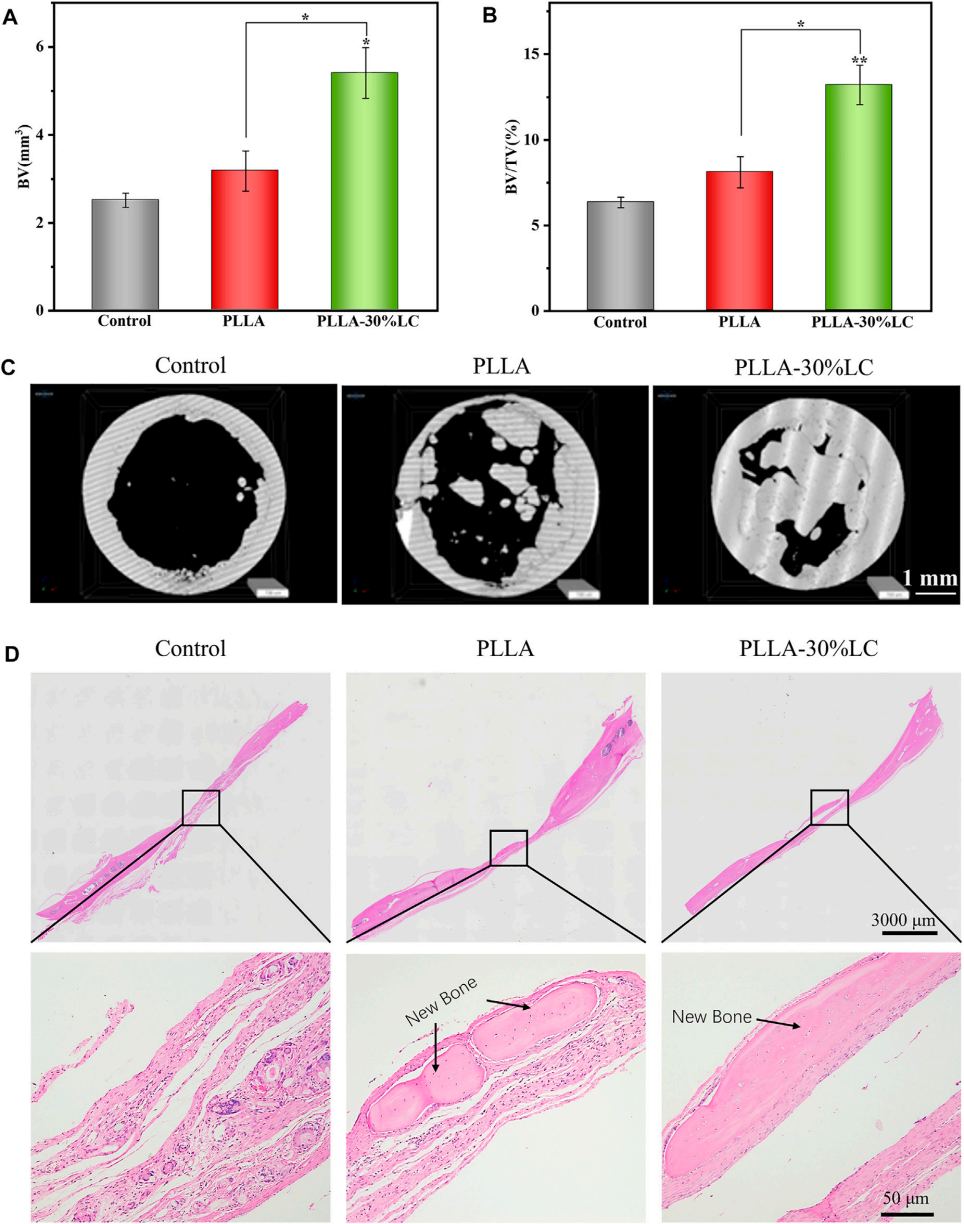
**(A)** Bone volume analysis; **(B)** bone volume density analysis; **(C)** micro-CT image of an SD rat skull defect 4 weeks after operation; and **(D)** HE staining photograph of an SD rat skull defect.

According to the micro-CT analysis (Figure 7C), only a small amount of bone tissue was found at the edge of the defect area in the sham surgery group 4 weeks after surgery. This indicated that SD rats could not repair large-scale calvarial defects without other treatments in 4 weeks. Compared with the sham surgery (control) group, a small amount of new bone tissue could be seen in the PLLA group. This was because PLLA could promote cell proliferation and osteogenic differentiation at a certain level, but its bone repair ability was still weak. Compared with the control and PLLA groups, the PLLA-30% LC (experimental) group demonstrated excellent bone repair ability, with a large amount of new bone tissue generated in the defect area. The data on bone volume (Figure 7A) and bone volume density (Figure 7B) also showed that the bone repair effect of PLLA-30% LC was better than that of the other two groups. Subsequently, the H&E staining analysis (Figure 7D) 4 weeks after surgery showed that a large amount of new bone tissue was formed in the PLLA-30% LC group and a small amount of lamellar bone and woven bone was obvious in the PLLA group. However, the defect area was almost full of fibrous connective tissue, and no obvious new bone tissue formation was found in the control group. This may be when PLLA was modified with LC-30% CP liquid crystal, a liquid crystal layer could be formed on the surface of PLLA. The cholesteric group in the liquid crystal had biological activity and could promote cell adhesion, proliferation, and osteogenic differentiation, and remarkably improved the cell compatibility of the material. At the same time, it also improved the immunogenicity of PLLA via enhancing the M2 polarization of RAW264.7 which benefited the bone tissue regeneration *in vivo.* In addition, the LC layer was in the LC state at 37°C and could respond to changes in the external temperature, which may be conducive to the transportation of nutrients and the conduction of information *in vivo*. The special surface characteristics of liquid crystal were similar to the structure and function of the extracellular matrix, and more similar to the microenvironment of tissue culture *in vivo*.

## 4 Conclusion

This study showed that the LC phase domain distribution was uniform in the PLLA/LC composite films and the films were in the LC state at a physiological temperature. The LC phase in the PLLA/LC composite films was similar to the flow mosaic model of biological films with the property of flow and order that was conducive to material transportation and signal transmission and could respond to temperature stimuli sensitively. Cytocompatibility experiments showed that COC/CP LCs were beneficial to improve the cytocompatibility of PLLA. When the LC concentration was 30% (wt) and 50% (wt), the PLLA/LC composite films could significantly promote proliferation and adhesion. Compared with other methods of modifying the cell compatibility of PLLA, COC/CP LCs could functionalize the surface of PLLA without providing any specific biological functional ligands, thereby providing affinity for cell attachment. In addition, osteogenic immunomodulation experiments showed that the PLLA/LC composite films promoted the M2 polarization of RAW264.7, improved the inflammatory environment of implants, and promoted the osteogenic differentiation of BMSCs which were beneficial to the repair of bone tissue *in vivo*. Moreover, the PLLA/LC composite films were simpler to prepare, lower in cost, and easier to popularize. Therefore, this novel of LC modified biomaterials is very promising to be used in clinical bone defect treatment in the future.

## Data Availability

The original contributions presented in the study are included in the article/supplementary material, further inquiries can be directed to the corresponding authors.
